# The Role and Regulatory Mechanisms of Cuticular Wax in Crop Stress Tolerance and Yield

**DOI:** 10.3390/plants15040554

**Published:** 2026-02-10

**Authors:** Dezhi Han, Jiaming Lu, Caitong Zhao, Shahid Ali, Zhenfeng Jiang

**Affiliations:** 1 Heihe Branch of Heilongiiang Academy of Agricultural Sciences, No. 345 Huanchengxi Road, Heihe 164399, China; handezhi2008@163.com; 2 Key Laboratory of Agricultural Biological Functional Genes, College of Life Science, Northeast Agricultural University, Harbin 150030, China; m13609152079@163.com (J.L.); 18846773398@163.com (C.Z.); 3 Guangxi Key Laboratory of Agro-Environment and Agro-Products Safety, Key Laboratory of Crop Cultivation and Physiology, College of Agriculture, Guangxi University, Nanning 530004, China; shahidsafi926@gmail.com; 4 National Key Laboratory of Smart Farm Technology and System, National Research Center of Soybean Engineering and Technology, Northeast Agricultural University, Harbin 150030, China

**Keywords:** cuticle wax, crop stress tolerance, yield, regulatory network, genetic improvement

## Abstract

Cuticular waxes form a crucial hydrophobic barrier on the surface of aboveground organs in terrestrial plants. It strongly influences crop stress tolerance and yield stability, making them important target traits in modern crop breeding. This review systematically summarizes recent advances in how cuticular waxes contribute to crop stress tolerance and yield formation. It covers the chemical composition of cuticular waxes, key pathways and regulatory networks, and the physiological and biochemical mechanisms. In addition, this review highlights the role of cuticle waxes in maintaining crop yield and quality by regulating essential physiological processes, including photosynthetic metabolism and water-use efficiency. Current research indicates that cuticular wax accumulation shows strong crop-specific patterns and is dynamically regulated by environmental factors. Breakthrough studies in major crops have clarified the regulatory mechanisms of several core genes and demonstrated that cuticular waxes enhance stress resistance by strengthening physical barriers, improving water-use efficiency, and protecting photosynthetic structures. A deeper understanding of cuticular wax regulatory mechanisms will help reveal the molecular basis of crop stress resistance and provide both theoretical support and practical guidance for breeding crop varieties with enhanced stress tolerance and stable yields.

## 1. Introduction

The cuticle is a hydrophobic barrier covering the surface of aboveground plant organs, primarily composed of cutin, cuticular waxes, and cuticle membranes. Its structural integrity and compositional complexity directly influence material exchange and signal transduction between plants and their external environment [1]. The epidermal cells on the surface of the cuticle layer are tightly packed, with stomata and trichomes interspersed throughout. Waxy crystals deposit on the outer layer of epidermal cells in tubular, lamellar, or granular forms, creating an additional physical protective layer [2]. Cuticular waxes are hydrophobic lipid mixtures that coat the surfaces of plant organs and play an essential role in enabling plants to adapt to terrestrial environments [3]. Through long-term evolution, cuticular wax has been shown to reduce non-stomatal water loss; protect plants from ultraviolet radiation and mechanical damage; and contribute critically to crops’ responses to abiotic stresses such as drought, salinity, and extreme temperatures, as well as to biotic stresses including pests and diseases [4]. As global climate change intensifies, the negative effects of environmental stress on crop yields have become increasingly evident. Consequently, regulating the synthesis and accumulation of cuticular wax to enhance crop stress tolerance and ensure yield stability has emerged as a major focus in agricultural research [5].

In recent years, with the advancement of technologies such as high-throughput sequencing, lipidomics, and gene editing, researchers have identified numerous key genes and transcription factors involved in wax biosynthesis, transport, and regulation in model plants and various crops. This has progressively revealed the central role of wax in plant adaptation to stress and its intricate regulatory network [4,6,7]. Extensive research indicates that cuticular waxes participate in crop stress responses through multiple mechanisms. Under abiotic stress conditions, cuticular waxes enhance drought tolerance by reducing water loss and improve resistance to salinity and extreme temperatures by modulating lipid composition [8]. In the context of biotic stress, not only do cuticular waxes function as physical barriers to limit pathogen invasion and insect feeding but also specific wax components act as chemical signals that trigger the expression of defense-related genes [9]. Beyond their role in stress responses, cuticular waxes indirectly influence crop yield and quality by regulating key physiological processes, including photosynthetic efficiency and water-use efficiency [10]. Under adverse conditions, crops with higher cuticular wax content tend to maintain more stable physiological metabolism, thereby reducing yield losses and preserving desirable agronomic traits [11].

Given the pivotal role of cuticular waxes in plant stress resistance, genetic manipulation of their biosynthesis and accumulation has emerged as a promising strategy for breeding crop varieties that are water-efficient; drought-tolerant; disease-resistant; and capable of achieving high, stable yields. This review systematically examines the chemical composition of cuticular waxes, their biosynthetic and transport pathways, the core component of their regulatory networks, their mechanisms of action in stress responses, and their effects on crop yield and quality. It aims to provide theoretical support for the genetic improvement of crop stress tolerance and practical guidance for high-yield, high-quality crop production.

## 2. The Main Chemical Components of Cuticular Waxes and Their Biosynthesis and Transport Pathways

Cuticular waxes are primarily composed of very long-chain fatty acids (VLCFAs) and their derivatives, including alkanes, primary and secondary alcohols, aldehydes, ketones, esters, triterpenoids, and certain secondary metabolites. The composition and abundance of these components vary markedly among plant species, organs, and growth conditions (Table 1) [12]. 

VLCFAs are processed through two major biosynthetic routes. In the acyl reduction pathway, VLCFAs give rise to even-carbon-chain primary alcohols and esters. In contrast, the decarboxylation pathway produces even-carbon-chain aldehydes and odd-carbon-chain alkanes. In the stem epidermis of Arabidopsis thaliana, alkanes can be further converted into secondary alcohols and ketones, whereas cuticular waxes of monocotyledonous crops such as rice lack secondary alcohols or ketones [24,25]. Consequently, alkanes are considered the terminal products of the decarboxylation pathway in monocotyledons [25]. Notably, Zhang et al. identified a hydroxylase involved in the formation of ultra-long odd-chain primary alcohols in rice, filling a critical gap in our understanding of cuticle wax biosynthesis in monocot crops [26].

Studies using the dicot model plant Arabidopsis thaliana have demonstrated that cuticular wax biosynthesis occurs primarily in the endoplasmic reticulum of epidermal cells [27]. Fatty acid synthesis begins in plastids, where acetyl-CoA is converted into C16 and C18 saturated fatty acids by fatty acid synthase (FAS). These precursors are then transported to the endoplasmic reticulum, where they are elongated into VLCFAs by the fatty acid elongation (FAE) complex. This elongation cycle involves a series of reactions catalyzed by β-ketoacyl-CoA reductase (KCR), β-hydroxyacyl-CoA dehydratase (HCD), and trans-acyl-CoA reductase (TCR), ensuring the stepwise extension of fatty acid chains.

Following their synthesis, VLCFAs are modified through two principal pathways to generate distinct wax components. In the acyl reduction pathway, VLCFAs are reduced to primary alcohols by fatty acyl coenzyme A reductase (FAR), which subsequently esterifies with fatty acids by wax ester synthase/diacylglycerol transferase 1 (WSD1) to form wax esters [28,29]. In the decarboxylation pathway, VLCFAs are first converted into aldehydes, which are then transformed into alkanes through the action of the CER1/CER3 complex. In dicot species, these alkanes can be modified by endo-hydroxylation catalyzed by the cytochrome P450 monooxygenase MAH1 (CYP96A15), resulting in the formation of secondary alcohols and ketones [27]. Additionally, as key components of plant cuticular waxes, triterpenoid compounds are synthesized via the mevalonate pathway. Through the catalysis of enzymes such as terpenoid synthase and cytochrome P450 monooxygenase, they are progressively formed [30].

Once synthesized, cuticular wax components are transported from the endoplasmic reticulum to the plasma membrane (PM) through the Golgi apparatus and trans-Golgi network (TGN). Their export to the epidermis surface is mediated by plasma membrane-localized ATP-binding cassette G (ABCG) subfamily transporters and lipid transporter proteins (LTPs) [12]. ABCG transporters, such as ABCG12 in Arabidopsis and TaABCG in wheat, actively export precursors across the plasma membrane into the apoplastic space [4,21]. In parallel, LTPs, including GhLTP4 in cotton and ZmLTP in maize, bind hydrophobic wax components in the apoplast and facilitate their movement and deposition in the cuticle [31,32].

## 3. Regulatory Network of Cuticular Wax Synthesis

The biosynthesis of cuticular wax is tightly regulated at multiple levels, including at the transcriptional [33], post-transcriptional [34,35], and post-translational [36] levels. This multilayered regulatory system allows cuticular wax synthesis to respond precisely to the requirements of plant growth and development, as well as to fluctuations in environmental conditions, thereby maintaining dynamic balance and adaptive regulation [5]. In the following sections, we outline the regulatory network governing cuticular wax biosynthesis, with a focus on the genetic regulation of key catalytic enzymes and functional genes, their transcriptional networks, and hormonal control mechanisms.

### 3.1. Key Catalytic Enzymes and Functional Genes

Cuticular wax biosynthesis relies on the coordinated activity of a series of key catalytic enzymes and functional genes. The expression and regulation of these genes directly determine the efficiency, composition, and accumulation of wax. Among them, components of the fatty acid elongation complex are critical determinants of both wax content and carbon chain length. For example, the apple HXXXD-type acyltransferase gene MdCER2L1 has been confirmed as an essential component of the fatty acid elongation complex. MdMYB106 transcriptionally activates its expression, whereas nitrogen availability negatively regulates cuticle wax synthesis by inducing MdBT2, which mediates ubiquitination and degradation of MdMYB106 [37].

Members of the KCS gene family exhibit strong substrate specificity and directly control the biosynthesis of VLCFAs with defined chain lengths. In wheat, TaKCS1 and TaKCS10 have been identified as key catalytic enzymes involved in cuticular wax biosynthesis [38,39]. Notably, not all KCS family members possess catalytic activity. In Arabidopsis, KCS3 lacks enzymatic activity but inhibits KCS6 by prolonging physical interactions among specific subunits of the elongation complex, thereby playing an important role in maintaining cuticular wax homeostasis [40].

The rate-limiting enzyme KCS6 is localized at endoplasmic reticulum–plasma membrane contact sites. Overexpression of VAP27-1 enhances interaction between the KCS6-CER2 complex, leading to increased VLCFA accumulation [41]. Furthermore, Yang et al. demonstrated that the CER2 protein coordinately regulated ACC1 and CER6/KCS6, enabling the extension of VLCFAs beyond C28 and providing new insights into the regulation of cuticular wax biosynthesis within the endoplasmic reticulum [42].

In the decarboxylation pathway, the CER1/CER3 complex functions as the key enzyme responsible for alkane biosynthesis, and the role of its homologs is highly conserved across plant species [14,16,43]. Meanwhile, the identification of crop-specific functional genes, such as ZmSRL5 in maize, W3 in wheat, and CsKCS20 in citrus, has provided important insight into the diversification of cuticular wax biosynthetic mechanisms among different crops [44,45,46].

In addition, cytochrome P450 enzymes in Arabidopsis, including CYP96A4, have been shown to participate in the biosynthesis of cuticular waxes and cutin monomers in leaves under wounding stress. These enzymes interact with the CER1/CER3 complex and modulate CER3 activity, thereby influencing aldehyde production [28]. Following synthesis, cuticular wax transport to the epidermal surface is also subject to strict regulation. For instance, silencing TaCER5, a member of the wheat ABCG transporter family, reduces wax accumulation and increases cuticle permeability in leaves, confirming its crucial role in cuticular wax transport [47].

Beyond the fatty acid elongation complex, wax-modifying enzymes, and transport-related genes, numerous additional functional genes contribute to the regulation of cuticular wax biosynthesis. The integration of high-throughput phenotyping and omics technologies has significantly accelerated the identification and functional analysis of transcription factors that regulate wax production. For example, in wheat, GWAS identified the key allele TaFAR5, which regulates leaf wax synthesis and drought resistance [48]. Genome-wide association studies and transcriptomic analyses of rapeseed have identified multiple SNPs and candidate genes significantly associated with leaf cuticular wax content, offering valuable targets for marker-assisted breeding [23,49]. Building on these findings, Lin et al. integrated multi-omics analyses to elucidate the relationship between cuticular wax metabolism and cuticle conductivity (GC) in maize. They identified high-molecular-weight wax esters as key predictors of GC and characterized 231 candidate genes associated with cuticular wax metabolism, providing an important genetic resource for maize breeding programs aimed at improving leaf wax traits [50]. Furthermore, CRISPR/Cas9 gene editing technology has emerged as a highly effective tool for improving crop wax traits. In a recent study, Lu et al. developed novel rice breeding materials through gene editing of the transcription factor NAT1, resulting in significantly increased cuticular wax accumulation that effectively prevented excessive water loss under high-temperature conditions [51]. Research findings indicate that editing key genes involved in wax synthesis holds significant potential for breeding superior new varieties.

### 3.2. Major Transcriptional Factors

The transcriptional regulation of cuticular wax biosynthesis is mediated by multiple transcription factors that bind to specific cis-regulatory elements within the promoter regions of cuticular wax-related genes, thereby activating or repressing their expression. Transcription factors (TFs) from several families participate in this regulatory process, with most belonging to three major plant TF families: APETALA2/ethylene response factor (AP2/ERF), Myeloblastosis (MYB), and homeodomain-lysine zipper IV (HD-Zip IV) [52].

Among these, the MYB family is the most extensively studied group of regulators of cuticular wax biosynthesis, particularly members of the R2R3-MYB subfamily. In rice, OsMYB60 acts as a positive regulator by directly binding to the OsCER1 promoter, thereby enhancing wax accumulation and improving drought tolerance [15]. In wheat, TaMYB60 promotes cuticular wax biosynthesis by activating the expression of TaFATB and TaCER1 [13]. Similarly, XsMYB30 in jujube positively regulates wax accumulation by binding to the promoters of genes involved in wax biosynthesis, wax transport, and fatty acid metabolism [53]. Extending these findings, Xu et al. demonstrated that XsMYB16 directly activates XsLTPG31 by targeting its promoter, thereby enhancing extracellular wax transport and promoting deposition in leaf epidermal cells [54].

The AP2/ERF family also plays a central role in regulating cuticular wax biosynthesis, particularly under stress conditions. In Arabidopsis, DREB26/ERF12 and its homologs, ERF13 and ERF14, positively regulate wax biosynthesis during drought stress by influencing VLC alkane accumulation and reducing leaf water loss [55]. However, this family also contains negative regulators. For example, Zhao et al. reported that CsRAP2-7 acts as a negative regulator in citrus by activating CsACO1-mediated ethylene biosynthesis, thereby indirectly suppressing cuticular wax synthesis and weakening drought tolerance [18].

The HD-ZIP IV family is specifically associated with epidermal development and cuticle formation. In wheat, TaHDG1.1 and TaHDG1.2 function as partially redundant positive regulators of cuticular wax biosynthesis. In contrast, TaCFL1 negatively regulates this process by attenuating TaHDG1-mediated transcriptional activation of the downstream gene TaKCS10 [39]. Precise manipulation of key transcription factors within the regulatory network controlling cuticular wax biosynthesis, particularly through genome-editing technologies, offers strong potential to optimize wax composition and structure. Such strategies may provide effective avenues for improving crop stress tolerance and yield stability.

### 3.3. Post-Transcriptional and Post-Translational Regulation

Previous studies have demonstrated that processes such as microRNA (miRNA)-mediated post-transcriptional regulation, post-translational modifications including protein phosphorylation and ubiquitination, and proteasome-mediated protein degradation all participate in regulating cuticular wax synthesis. This enables plants to respond more sensitively to environmental changes and internal signals, thereby finely balancing the relationship between wax synthesis, energy allocation, and growth and development [56].

MicroRNAs are endogenous non-coding small RNAs approximately 20–24 nucleotides in length that mediate post-transcriptional gene silencing by mediating cleavage or translational inhibition of target mRNAs [57]. Research has revealed that the expression level of osa-miR1848 negatively correlates with the mRNA abundance of the wax synthesis gene OsWS1. Furthermore, their expression patterns are opposite in rice leaves, panicles, and stems, as well as under drought stress. This indicates that OsWS1 is regulated by osa-miR1848 and participates in the dynamic accumulation of cuticular wax in rice to adapt to environmental changes [58]. In Arabidopsis, the regulatory mechanism of miRNA on wax synthesis has also been preliminarily explored. miR156 participates in the circadian regulation of wax synthesis-related genes by targeting the SPL9 transcription factor, providing potential targets for improving crop stress resistance and quality [20]. Looking ahead, systematically identifying differentially expressed miRNAs and their target genes in the wax synthesis pathway under stresses such as drought and low temperatures through high-throughput sequencing and degradomics will be a key approach to unraveling the complex regulatory network of wax in crops.

Protein modification, as a core mechanism of post-translational regulation, directly influences protein function by altering its structure, stability, or subcellular localization. Among these modifications, the roles of ubiquitination and phosphorylation in regulating cuticular wax synthesis in the epidermis have been most extensively studied [59,60]. Liu et al. revealed both the universality and specificity of protein phosphorylation modifications in the SCF complex-mediated ubiquitination and degradation of substrate proteins, providing novel insights for further investigation into the interactions between phosphorylation and ubiquitination modifications [61]. Among these, the ubiquitin–proteasome pathway is one of the most important mechanisms regulating protein stability. Research has revealed that the BTB-TAZ domain protein MdBT2 negatively regulates cuticular wax accumulation in apple by mediating the ubiquitination and degradation of the MYB transcription factor MdMYB106, thereby suppressing the expression of the cuticular wax synthesis gene MdCER2L1 [37]. In addition to ubiquitination, SUMOylation also participates in the regulation of wax synthesis in crops. The apple SUMO E3 ligase MdSIZ1 enhances protein stability by mediating SUMOylation of MdMYB30, thereby inhibiting its degradation via the 26S proteasome. This promotes MdKCS1 expression, positively regulating wax synthesis and further expanding the diversity of post-translational modifications in wax synthesis regulation [62]. Furthermore, research on protein phosphorylation in the direct regulation of wax synthase has provided clues indicating its significance. The leucine-rich repeat transmembrane protein kinase BM41 has been identified as a positive regulator of wax synthesis in sorghum. It activates KCS6 expression through phosphorylation, thereby promoting the accumulation of ultra-long-chain fatty acids and triterpenoid compounds in wax [63]. Zhao et al. revealed that the kinase PtrPPK1 directly interacts with PtrC2H2.2-6. Through phosphorylation modification, it promotes degradation of PtrC2H2.2-6 via the 26S proteasome, thereby releasing transcriptional repression and activating downstream synthetic pathways. Ultimately, this enhances poplar drought tolerance by regulating the accumulation of epidermal barrier substances [64]. In subsequent studies, systematically identifying the interacting proteins of transcription factors will help elucidate the molecular mechanisms by which different families of transcription factors regulate wax biosynthesis.

### 3.4. Regulation of Plant Hormones in Cuticular Wax Synthesis

Cuticular wax biosynthesis is coordinated by a complex hormonal network in which abscisic acid (ABA), jasmonic acid (JA), and ethylene (ET) play central roles. As vital endogenous signaling molecules, plant hormones integrate environmental cues and developmental programs, enabling plants to dynamically adjust the accumulation and composition of cuticular waxes in response to changing growth conditions [65].

ABA is one of the primary hormones regulating cuticular wax synthesis. Studies on ABA biosynthesis-deficient tomato mutants revealed that reduced ABA levels lead to pronounced thinning and structural abnormalities in the leaf cuticle, accompanied by a significant decrease in cuticular wax content [66]. In addition, ABA indirectly influences cuticular wax biosynthesis through crosstalk with other hormonal pathways. For example, melatonin suppresses ABA accumulation during mild drought conditions but promotes it under severe drought, thereby coordinately regulating the accumulation of C29 and C31 alkanes in watermelon cuticular wax [67].

Jasmonic acid (JA) and its derivatives, such as methyl jasmonate (MeJA), are also key hormones governing cuticular wax synthesis. In Arabidopsis, the JA signaling pathway core gene CYP96A4 participates in wound-induced cuticular wax biosynthesis by interacting with the CER1/CER3 enzyme complex [28]. Increasing evidence suggests that JA and ABA signaling pathways act synergistically to regulate cuticular wax biosynthesis. For instance, a study on sweet sorghum demonstrated that the combined exogenous application of ABA and JA increased cuticular wax content by 71.7% under drought stress, a significantly greater effect than that achieved with either hormone alone [68].

Ethylene (ET), as a gaseous hormone, regulates cuticular wax biosynthesis through a mechanism distinct from those of ABA and JA. In maize, ethylene was shown to activate the expression of the wax regulatory gene ZmERE and wax biosynthesis genes such as ZmGL1, ZmGL15, ZmFDH1, and ZmFAE1, thereby promoting wax accumulation in seedlings under normal and drought conditions [69]. In contrast, recent work in citrus identifies CsRAP2-7as a negative regulator of cuticular wax biosynthesis. This transcription factor suppresses wax accumulation by directly activating the ethylene biosynthetic gene CsACO1, leading to elevated ethylene levels [18]. Together, these findings indicate that ethylene exerts bidirectional effects on cuticular wax synthesis, which are likely dependent on species, hormone concentration, developmental stages, and stress intensity.

## 4. Mechanism of Cuticular Wax in Crop Stress Resistance

As the primary physicochemical barrier of the plant epidermis, cuticular wax contributes to crop stress resistance through multiple coordinated functions, including reducing non-stomatal water loss, maintaining membrane integrity, enhancing antioxidant capacity, regulating ion homeostasis, mediating stress signaling, and forming effective physicochemical defense barriers. Together, these functions provide the structural and functional foundations that enable crops to adapt to diverse environmental stresses [8,9].

### 4.1. Physiological Functions in Response to Abiotic Stress

**Figure 1:**
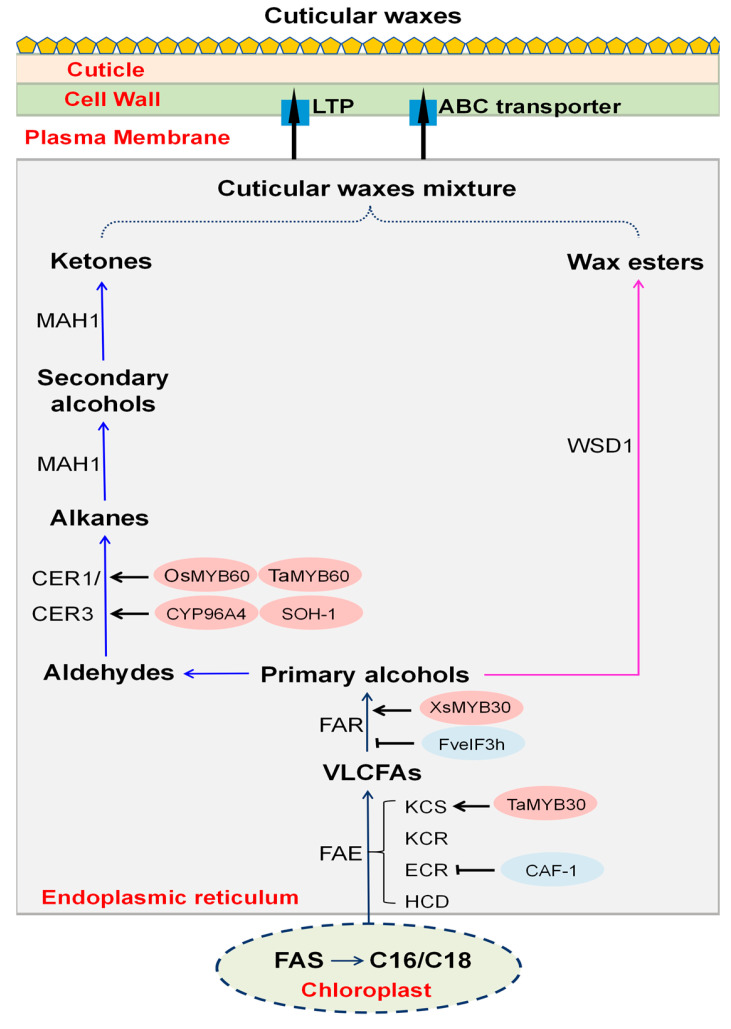
Schedule of cuticular wax synthesis. The red oval represents positive regulators of wax synthesis, while the blue oval represents negative regulators of wax synthesis. After VLCFAs are synthesized, they are mainly converted into different components of wax through two modification pathways. The blue arrows indicate the decarboxylation pathway, while the pink arrows indicate the acyl reduction pathway. Black arrows represent positive regulation, while black lines ending with a bar represent negative regulation.

Cuticular wax serves as the first line of defense between plants and their external environment and plays a crucial physiological role in crops’ responses to abiotic stresses such as drought, extreme temperatures, and salinity. Its core functions include reducing water loss, maintaining cellular osmotic pressure, restricting the entry of harmful substances, and protecting cellular structural integrity (Figure 1).

Numerous strong, positive correlations between cuticular wax accumulation and drought tolerance have been documented in many crops. For example, overexpression of XsMYB30 in poplar increases leaf wax deposition and significantly enhances drought tolerance [53]. In wheat, systematic analysis of natural populations revealed that variation in the TaFAR5-TaFAR3 module confers drought tolerance by regulating leaf cuticular wax biosynthesis [48]. Similarly, Zhao et al. identified the PtrPPK1–PtrC2H2.2 6–PtrCYP86A7/A8 regulatory pathway in poplar, showing that phosphorylation-dependent degradation of PtrC2H2.2-6 alleviates repression of wax biosynthesis genes, thereby enhancing leaf water retention and drought stress [64].

Beyond total wax content, wax composition and its physical structure are also critical determinants of drought resistance. In maize, mutation of ZmSRL5 does not significantly alter total leaf wax content but alters wax crystal structure, leading to accelerated water loss and reduced drought tolerance [44]. In tobacco, mutants deficient in alkanes fail to effectively reduce transpiration under drought conditions, resulting in leaf death and poor recovery capacity, whereas mutants lacking fatty alcohols exhibit higher drought tolerance [70]. Consistently, Li et al. demonstrated that increased levels of VLC alkanes produced through CER3-CER1 interactions reduced water loss, whereas increased VLC 1-alcohols formed via CER3-SOH1 interactions enhanced cuticular transpiration. These findings highlight how specific wax components modulate cuticle properties and drought resistance [71].

Cuticular wax also plays a critical role in protecting crops from temperature extremes. Under cold stress, Arabidopsis wax-deficient mutant cer3-6 freezes at higher temperatures, whereas the wax-overproducing mutant dewax exhibits a lower freezing point [8]. Combined gas chromatography–mass spectrometry and Fourier transform infrared spectroscopy analysis have shown that specific wax components synergistically reduce non-stomatal water loss and inhibit ice crystal formation, thereby enhancing frost resistance and water retention [8]. Similar mechanisms have been observed in wheat, where exposure to low temperatures (4 °C) rapidly induced TaCER1-1A expression, increasing n-alkane accumulation and cuticle hydrophobicity to mitigate cold-induced cellular dehydration [72].

Under high-temperature and ultraviolet radiation stress, the cuticle protects crops by absorbing and reflecting harmful radiation that would otherwise damage DNA, proteins, and photosynthetic pigments. In wheat, Wang et al. reported that TaHDA19 suppresses TaMYB30 expression by histone deacetylation, thereby inhibiting cuticular wax biosynthesis. Upon UV-B exposure, activation of the UV resistance locus 8 (UVR8) induces TaHY, which relieves TaHDA19-mediated repression of TaMYB30 and promotes cuticular wax accumulation [73].

Research shows that waxy traits are shaped by both heritable genetic factors and dynamic environmental responses. For example, Medicago ruthenica exhibits heritable, environmentally driven waxy adaptations [74], while perennial alpine herbs rely on phenotypic plasticity to cope with climate change [75]. Dwarf wormwood shows elevated cuticular wax content at both high altitudes (4200 m), where UV-B radiation is intense, and lower altitudes (2600 m), where UV exposure is moderate, indicating that elevated wax deposition is a common adaptive response in high-altitude environments [75].

Under salinity stress, crops experience both osmotic stress and ion toxicity, and cuticular waxes mitigate these effects through several mechanisms. First, wax forms a hydrophobic barrier that restricts the entry of salt ions through epidermal cells. For example, Arabidopsis plants overexpressing apple MdLACS4 show increased cuticular wax accumulation and enhanced salt tolerance [76]. Similarly, KCS19 knockout mutants exhibit reduced wax and alkane content and decreased salt tolerances, whereas KCS19 overexpression lines display enhanced wax accumulation and salt resistance [77]. Second, cuticular wax contributes to salt tolerance by influencing ion transport and excretion. In Suaeda chinenis, osmotic stress reduces epidermal permeability while increasing total wax content and alkane accumulation, accompanied by upregulation of genes involved in wax biosynthesis and transport [21]. Third, wax accumulation can alleviate oxidative damage induced by salt stress. In Arabidopsis, the basal domain regulator 3 (BDR3) loss-of-function mutant tos1 increases accumulation, reduces water loss and ROS accumulation, and thereby enhances salt tolerance [78].

However, not all studies attribute stress tolerance primarily to cuticular wax. In barley, osmotic stress induces root-to-stem signaling that upregulates leaf wax and cutin-related gene expression and increases their accumulation. Yet, barley stomatal conductance rapidly and significantly decreases, while cuticular wax conductance remains unaffected. This suggests that under osmotic stress, stomatal regulation is more crucial for preventing water loss than cuticular wax regulation or adaptation [79]. These findings underscore the importance of considering the coordinated contribution of both stomatal and non-stomatal pathways when evaluating the role of cuticular wax in crop stress tolerance.

### 4.2. Physical and Chemical Barriers Against Biotic Stress

In addition to their protective role against abiotic stresses, cuticular waxes function as both physical barriers and chemical regulators in plant defenses against biotic stresses, including pathogens and herbivorous insects. Physical obstruction represents the most direct mechanism by which cuticular waxes combat biotic stresses in plants. The protruding micro- and nanostructures formed by cuticular wax crystals reduce surface smoothness and effectively hinder the attachment and penetration of dust particles, pathogen spores, and insect mouthparts on plant surfaces [80].

Accumulating evidence suggests that the integrity and permeability of the cuticular wax layer strongly influence the survival, colonization, and feeding behavior of bacterial pathogens and piercing-sucking insects. Altered cuticle permeability can either enhance resistance or increase susceptibility, depending on the biological context [81]. For example, Lorrai et al. showed that disruption of pectin integrity in Arabidopsis increases cuticle permeability and promotes the accumulation of antimicrobial compounds on the leaf surface, resulting in near-complete resistance to Botrytis cinerea [82]. Similarly, Chen et al. identified the tobacco cuticle-related factor NtCRF, which is induced by whitefly infection and positively regulates resistance to whiteflies and peach aphids. Silencing NtCRF disrupts the structural integrity of leaf cuticles and increases epidermal permeability, thereby shortening the duration of phloem feeding by piercing-sucking insects [83].

In vascular plants, cuticular waxes cover the surfaces of aerial primary tissues and typically form continuous two-dimensional films. In contrast, plant surfaces bearing three-dimensional cuticular wax coverage may inhibit the attachment, movement, and foraging behavior of insect predators and parasitoid wasps [84]. Experimental evidence supports this view: onion-mustard stems covered with three-dimensional cuticular wax notably reduced the visitation frequency, movement distance, and running speed of black hairy ants, whereas smooth stems lacking cuticular wax and trichomes showed no such inhibitory effect [85]. Analysis by Abbas et al. of six citrus varieties revealed that cuticular wax content alone accounted for 33.5% of the variation in citrus leafminer larvae density, while other chemical components, such as aldehydes and alcohols, showed no significant effect [86].

**Figure 2:**
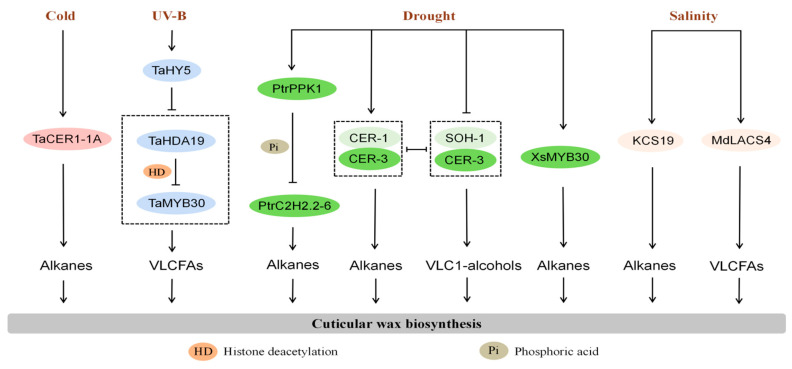
Mechanisms of key wax synthesis and regulatory genes in responding to abiotic stress. Black arrows represent positive regulation, while black lines ending with a bar represent negative regulation.

Chemical defense represents another crucial mechanism by which cuticular waxes contribute to biotic stress resistance. Specific wax components can directly inhibit pathogen growth and reproduction, act as repellents, or exert toxic effects on pests (Figure 2). In maize, the cuticular was biosynthetic gene ZmCER1 has been shown to influence plant defense pathways. GO and KEGG analyses revealed that genes differentially expressed in this gene were enriched in pathways encompassing disease processes, benzothiazine biosynthesis, and riboflavin metabolism. This evidence indicates that the ZmCER1 gene contributes to the construction of plant defense systems against diseases and pests through multiple pathways [14].

Powdery mildew caused by Blumeria graminis is a major disease affecting barley and wheat. Increasing evidence indicates that this pathogen recognizes and utilizes specific components of plant cuticular waxes to initiate its pre-infection processes, such as conidia germination and appressorium development. The wheat chromatin remodeling protein TaSWP73 is known to regulate post-penetration resistance against powdery mildew. Inhibiting the TaSARD1-TaICS1 module in the salicylic acid synthesis pathway reduces salicylic acid biosynthesis, thereby mediating the affinity interaction between wheat and powdery mildew [87]. Similarly, the chromatin-associated factor CAF-1 epigenetically suppresses the cuticular wax biosynthesis gene TaECR and the salicylic acid synthesis-activating gene TaSARD1, thereby negatively regulating cuticular wax and salicylic acid synthesis and finely modulating wheat susceptibility to powdery mildew [88]. Meanwhile, the synergistic insecticidal effects of cuticular waxes and plant hormones have been extensively studied. Liu et al. further showed that deletion of the maize cuticular wax synthesis gene ZmGL8 activates the JA signaling pathway, thereby enhancing maize’s chemical resistance to herbivores. In this process, lipid metabolism related to very long-chain fatty acids (VLCFAs) plays a pivotal role in regulating the trade-off between cuticular waxes and JA-mediated chemical defense in plants [9]. It is worth noting that while abscisic acid (ABA) generally suppresses plant immunity against Botrytis cinerea in most current studies, Cui et al. discovered that the resistance conferred by cuticle permeability in Arabidopsis can be genetically decoupled from ABA sensitivity. However, its immune function requires the negative regulation of a parallel ABA-dependent cell death pathway [89].

## 5. Effects of Cuticular Wax on Crop Yield and Quality

Cuticular waxes influence multiple aspects of crop yield and quality, including photosynthetic efficiency, water-use efficiency, nutrient uptake and utilization, and resistance to pests and diseases. By regulating the exchange of materials and energy between plants and their environment, cuticular waxes directly or indirectly influence crop growth and development, and the accumulation and distribution of photosynthetic products, ultimately affecting yield potential and quality traits [90].

### 5.1. Effects on Photosynthesis and Water-Use Efficiency

Under abiotic stress, such as drought, cuticular wax plays a critical role in sustaining photosynthesis and improving water-use efficiency (WUE) by limiting non-stomatal water loss. As soil moisture declines, stomatal closure becomes the primary response to reduce transpiration, shifting water loss predominantly to non-stomatal pathways. A thick and structurally intact cuticular wax layer effectively restricts cuticular transpiration, thereby maintaining leaf water status and cellular hydration [22]. By conserving water at the leaf surface, enhanced cuticular deposition can delay complete stomatal closure and extend the duration of active photosynthesis during drought.

Physiological studies support this protective role of cuticular wax. In wheat near-isogenic lines, waxy genotypes exhibit higher photosynthetic rates, leaf water potential, and relative water content under drought stress. One of the key reasons for their higher yields compared to non-waxy lines lies in these characteristics [10]. A study revealed that the rice OsMYB60 mutant displayed reduced drought tolerance, along with increased chlorophyll leakage and water loss rates. qRT-PCR results indicated downregulated expression of cuticular wax biosynthesis-related genes, resulting in lower total cuticular wax content on rice leaves under normal growth conditions [15]. Overexpression of FveIF3h in strawberry led to decreased cuticular wax content, reduced leaf chlorophyll content, increased osmotic permeability, and impaired drought tolerance [91]. It is noteworthy that several plant species have been observed to accumulate higher levels of cuticular wax under light exposure than in darkness [6,92,93]. However, Shellakkutti et al. found that although barley exhibited increased cuticular wax content under osmotic stress, there was no marked change in cuticular transpiration rates, indicating that stomatal regulation remains the primary factor controlling water loss [79].

Water-use efficiency is one of the key factors influencing crop yield formation. The mechanism by which cuticular wax enhances crop water-use efficiency primarily involves its hydrophobic properties. Extensive research indicates a positive correlation between cuticular wax content and crop water-use efficiency. Analysis of the citrus mutant MT revealed that increased accumulation of aliphatic cuticular wax reduces leaf cuticle permeability, ultimately enhancing drought tolerance and water-use efficiency in mutant plants [94]. Arabidopsis DREB26OX plants also reduce water loss by increasing cuticular wax accumulation, resulting in a substantial improvement in water-use efficiency [55]. Moreover, cuticular wax content and composition differ among crop varieties, leading to varied effects on water-use efficiency. The analysis of cuticular wax composition in watermelon M20 leaves under drought stress revealed significant induction of C29 and C31 alkanes. M20 exhibited lower water loss rates and stronger drought tolerance, suggesting that alkane carbon chain length may influence crop water regulation [67]. Li et al.’s research supports this argument: C31 alkanes accounted for more than 75% of total alkanes across all stress treatment conditions. Following stress treatment, both water loss rate and chlorophyll leaching rate in Bawang leaves decreased, exhibiting a significant negative correlation with C31 alkane content [95]. To confirm the pivotal role of alkanes in cuticular wax composition, Negin et al. studied an alkane-deficient tobacco mutant. They found it failed to effectively reduce transpiration rates, leading to leaf necrosis, impaired recovery capacity, and stem cracking, resulting in significant yield losses. The root cause lies in alkane cuticular waxes, which enable crops to seal the cuticle after stomatal closure further. This reduces leaf necrosis and accelerates post-drought recovery. Conversely, crops lacking cuticular waxes containing fatty alcohols exhibit enhanced drought tolerance [70].

### 5.2. Contributions to Maintaining Stable Crop Yields and Quality Under Adverse Conditions

As a key functional layer of crop epidermis, the cuticular wax plays a vital role in crop yield formation and quality assurance under stress conditions through multiple pathways. These include enhancing water-use efficiency, strengthening stress-resistant defense mechanisms, and regulating photosynthetic metabolic balance. This conclusion has been widely validated across diverse crop groups, including cereal, fruit, and oilseed crops [4,14,94]. Extensive research shows that increased cuticular wax content or enrichment of specific components under abiotic stress can provide a stable internal environment for crop growth and yield formation by reducing non-transpiratory water loss and mitigating damage from biotic and abiotic stresses. This effect is particularly pronounced under water-limited or environmental stresses such as drought and high temperatures.

Among cereal crops, the flowering and grain-filling stages are the most sensitive to water stress. At this time, the senescence of functional leaves, particularly the flag leaf, and the decline in photosynthetic capacity will directly lead to insufficient grain filling and reduced thousand-grain weight. Research indicates that varieties with intact cuticular wax layers better withstand post-flowering drought by prolonging leaf function. This ensures a sustained supply of photosynthetic products to grains, thereby maintaining relatively stable yields. Abundant field and simulated trial evidence supports this view. In rice, lines overexpressing cuticular wax synthesis genes such as OsGL1-2 or OsCUT1 exhibit markedly enhanced drought tolerance, characterized by lower epidermal permeability, reduced water loss rates, and relatively higher survival rates [96,97]. Likewise, the disease-causing gene ZmCER1 in the corn leaf-wrinkling mutant rgd4 is involved in cuticular wax synthesis. Its functional deficiency leads to reduced cuticular wax density on leaves, triggering multiple phenotypes, including plant dwarfism, abnormal leaf shape, and reduced pollen fertility, directly impacting crop growth and development as well as final yield formation [14]. In a recent study, Liu et al. conducted a genome-wide association analysis and discovered that allelic variation in wheat TaFAR5 promotes the synthesis of the cuticular wax component p-anisaldehyde by regulating its expression and interacting with TaFAR3. Knockout of the TaFAR5 gene reduces drought tolerance in wheat seedlings. The TaFAR5 dominant allele exhibits a broader distribution in tetraploid wheat and a higher frequency in arid regions, indicating its adaptive value under natural selection and in artificial domestication. This implies that the protective role of cuticular waxes in grain crop yield may be genetically specific [48].

Beyond contributing to grain yield, cuticular waxes exert a decisive impact on agricultural product quality, particularly the post-harvest quality and cosmetic appearance of fruits and vegetables. Current research confirms that cuticular waxes function as critical barriers against water and solutes, regulating crop gas exchange processes when stomata are closed or absent [98]. Due to variations in cuticular wax across different species, aquatic plants and terrestrial plants exhibit differing capacities for carbon dioxide and oxygen exchange [99]. Compared to varieties with thinner wax layers, crops with higher cuticular wax content exhibit less water loss and longer storage life [100]. For example, variations in apple skin characteristics correspond to different cuticular wax compositions. The glossy quality of apple fruit largely depends on the alcohol compounds within the cuticular wax [101]. The sticky texture observed on apple surfaces during storage is primarily associated with fluctuations in the content of secondary alcohols [102], and the content of alkanes in cuticular wax shows a decreasing trend, while the content of fatty acids increased [103]. In tomatoes, the Woolly gene influences cuticular wax synthesis by cooperatively regulating genes such as SlCER6 with the SlMYB31 transcription factor, potentially affecting fruit shelf life and disease resistance [104]. The strawberry translation initiation factor FveIF3h negatively regulates leaf cuticular wax content by restraining the translation of its synthesis protein CER4/FAR3 through an upstream open reading frame (uORF). Plants overexpressing FveIF3h exhibit increased epidermal permeability and reduced drought tolerance, offering a novel approach to modulating wax content at the translational level for enhancing fruit quality or stress resistance [91].

## 6. Summary and Outlook

**Figure 3:**
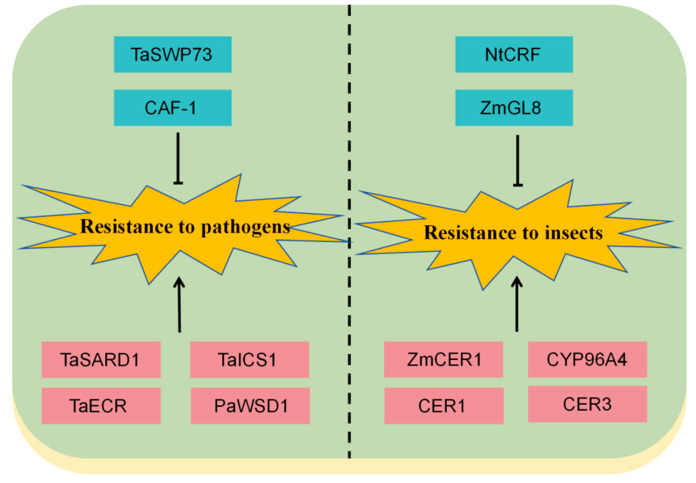
Mechanism of key wax synthesis and regulatory genes in responding to biotic stress. Red rectangles represent positive regulatory factors in response to biotic stress, while blue rectangles represent negative regulatory factors in response to biotic stress. Black arrows represent positive regulation, while black lines ending with a bar represent negative regulation.

As a crucial hydrophobic protective barrier in the epidermis, the biosynthesis, regulatory networks, and stress-resistant functions of plant cuticular wax have become central research topics in plant stress tolerance and breeding studies under the backdrop of global climate change. In recent years, a series of breakthrough studies have not only refined the molecular pathways of cuticular wax synthesis but also revealed its cross-interactions with multiple signaling pathways and adaptive mechanisms under various abiotic stresses (Figure 3). Regarding cuticular wax synthesis mechanisms, the two-step reduction pathway involving CER3 and SOH1 in Arabidopsis has established a novel route for primary alcohol synthesis. The core SOH1-CER3-CER1 module achieves precise regulation of the ratio between alkane and primary alcohol synthesis through competitive binding, providing the first molecular-level evidence of the close connection between the synthesis pathways of major cuticular wax components [71]. In terms of wax regulation networks, different species have revealed distinct pathways for cuticular wax synthesis. In rice, TT2 establishes a novel regulatory pathway independent of traditional heat tolerance mechanisms by integrating G protein signaling, calcium signaling, and wax metabolism. The stable expression of OsWR2 mediated by this pathway correlates with consistent cuticular wax content, making it a key factor in enhancing rice heat tolerance [105]. The PtrPPK1-PtrC2H2.2-6-PtrCYP86A7/A8 pathway in poplar reveals a key mechanism for alleviating the inhibition of cuticular wax synthesis through dephosphorylation in forest trees, thereby enriching the research framework for cuticular wax regulation in woody plants [64].

In the field of systems biology, integrating multi-omics data enables comprehensive analysis of the regulatory network governing cuticular wax synthesis and stress resistance mechanisms, offering novel insights for precision improvement of wax traits in crops. Multi-omics integrated analysis has successfully identified multiple novel genes and regulatory factors associated with wax synthesis. Through integrated transcriptomic and metabolomic analysis, Sumbur et al. successfully identified five key wax synthesis enzyme genes and one ABC transporter gene in Saussurea costus [21]. Chen et al. combined genomics and protein interaction analysis to discover that TaCFL1 negatively regulates wax synthesis in wheat by suppressing TaKCS10 expression mediated by TaHDG1 [39]. In studies of combined stress responses, systems biology approaches can reveal the regulatory mechanisms of waxes under multiple stresses. In a recent study, through genome-wide association analysis and transgenic validation, Liu et al. discovered that the regulatory modules of wheat TaFAR5 and TaFAR3 exhibit significantly enhanced synergistic control over wax synthesis and the ABA signaling pathway under combined drought and salt stress conditions. This interaction promotes the synthesis of extra-long-chain fatty alcohols in waxes and enhances stress resistance [48]. In the future, with the advancement of technologies such as single-cell omics and spatial transcriptomics, systems biology approaches are expected to precisely elucidate the cell-specific and spatiotemporal dynamic regulatory mechanisms underlying cuticular wax synthesis. This will provide more precise targets for the directed improvement of cuticular wax traits.

Despite remarkable progress in current research, the synergistic or antagonistic interactions between G proteins, calcium signaling, and hormone signaling in cuticular wax metabolism, as well as regulatory mechanisms involving epigenetic modifications and post-translational control, remain incompletely elucidated. Furthermore, existing studies on the role of cuticular wax in crop stress resistance predominantly focus on drought stress, lacking validation across multiple stress types. Key genes identified in signaling pathway studies offer exciting potential targets for plant breeding and gene editing [105,106], providing novel strategies for improving crop stress tolerance through cuticular waxes. Looking ahead, the regulation of crop cuticular wax represents a key direction in horticultural crop quality breeding. By leveraging a deep understanding of the cuticular wax synthesis pathway and synthetic biology strategies to reconstruct or optimize specific component-synthesis modules in crops, we can create germplasm resources with novel stress-tolerant traits. Genetic approaches to improving crop cuticular wax traits—such as identifying key genes and SNPs involved in cuticular wax synthesis through combined GWAS and transcriptomic analyses [107], or precisely regulating wax synthesis pathways in crops like wheat [108] and maize [109] via gene editing technologies—have become crucial strategies for developing high-yielding, water-efficient, stress-tolerant, and high-quality new varieties. Through multidisciplinary integration, this research aims to uncover novel functions and mechanisms of cuticular waxes in modern agricultural environments. By elucidating these processes, it is expected to provide robust theoretical support for breeding new crop varieties that are resource-efficient, environmentally adaptable, and capable of delivering both high yields and superior quality. Such advancements hold significant implications for effectively addressing global climate change, ensuring food security, and promoting sustainable agricultural development.

## Figures and Tables

**Table 1:** Cuticular wax components of major plant species.

Plant Species	Primary Wax Components	Characteristic Components
*Triticum aestivum*	Primary alcohols, alkanes, aldehydes	C26–C30 primary alcohols, C33 alkanes [10,13]
*Zea mays*	Alkanes, triterpenoids, fatty acids	C28–C32 alkanes, β-diketones [9,14]
*Oryza sativa*	Alkanes, primary alcohols, esters	C24–C28 alkanes, C26 primary alcohol [15,16]
*Citrus sinensis*	Primary alcohols, esters, aldehydes	C24–C28 primary alcohols, wax esters [17,18]
*Arabidopsis thaliana*	Alkanes, secondary alcohols, ketones	C29 alkanes, C29 secondary alcohols [19,20]
*Ammopiptanthus mongolicus*	Alkanes, fatty acids	C28–C32 alkanes [21]
*Malus domestica*	Primary alcohols, alkanes, esters	C26–C30 primary alcohols, wax esters [22]
*Brassica napus*	Alkanes, primary alcohols, fatty acids	C28–C30 alkanes, C26 primary alcohols [23]

## Data Availability

No new data were created or analyzed in this study.
